# The Role of Efflux Pumps in *Schistosoma mansoni* Praziquantel Resistant Phenotype

**DOI:** 10.1371/journal.pone.0140147

**Published:** 2015-10-07

**Authors:** António Pinto-Almeida, Tiago Mendes, Ana Armada, Silvana Belo, Emanuel Carrilho, Miguel Viveiros, Ana Afonso

**Affiliations:** 1 Graduate Program in Areas of Basic and Applied Biology, Instituto de Ciências Biomédicas Abel Salazar, Universidade do Porto, Porto, Portugal; 2 Medical Parasitology Unit, Global Health and Tropical Medicine, GHTM, Instituto de Higiene e Medicina Tropical, IHMT, Universidade Nova de Lisboa, UNL, Lisbon, Portugal; 3 Universidade de São Paulo, Instituto de Química de São Carlos, São Carlos, SP, Brazil; 4 Institute of Biology, Universidade de Campinas, Campinas, SP, Brazil; 5 Medical Microbiology Unit, Global Health and Tropical Medicine, GHTM, Instituto de Higiene e Medicina Tropical, IHMT, Universidade Nova de Lisboa, UNL, Lisbon, Portugal; 6 Laboratory of Parasitology, Departamento de Morfologia e Patologia, Universidade Federal de São Carlos, São Carlos, SP, Brazil; Queensland Institute of Medical Research, AUSTRALIA

## Abstract

**Background:**

Schistosomiasis is a neglected disease caused by a trematode of the genus *Schistosoma* that is second only to malaria in public health significance in Africa, South America, and Asia. Praziquantel (PZQ) is the drug of choice to treat this disease due to its high cure rates and no significant side effects. However, in the last years increasingly cases of tolerance to PZQ have been reported, which has caused growing concerns regarding the emergency of resistance to this drug.

**Methodology/Principal Findings:**

Here we describe the selection of a parasitic strain that has a stable resistance phenotype to PZQ. It has been reported that drug resistance in helminths might involve efflux pumps such as members of ATP-binding cassette transport proteins, including P-glycoprotein and multidrug resistance-associated protein families. Here we evaluate the role of efflux pumps in *Schistosoma mansoni* resistance to PZQ, by comparing the efflux pumps activity in susceptible and resistant strains. The evaluation of the efflux activity was performed by an ethidium bromide accumulation assay in presence and absence of Verapamil. The role of efflux pumps in resistance to PZQ was further investigated comparing the response of susceptible and resistant parasites in the absence and presence of different doses of Verapamil, in an *ex vivo* assay, and these results were further reinforced through the comparison of the expression levels of *SmMDR2* RNA by RT-PCR.

**Conclusions/Significance:**

This work strongly suggests the involvement of Pgp-like transporters SMDR2 in Praziquantel drug resistance in *S*. *mansoni*. Low doses of Verapamil successfully reverted drug resistance. Our results might give an indication that a combination therapy with PZQ and natural or synthetic Pgp modulators can be an effective strategy for the treatment of confirmed cases of resistance to PZQ in *S*. *mansoni*.

## Introduction

Schistosomiasis is a neglected tropical disease that affects approximately 249 million people worldwide, 97% of which are located on the African continent. It ranks, with malaria and tuberculosis, as a major source of morbidity despite strenuous control efforts [1, 2]. Furthermore, amongst all the parasitic diseases, schistosomiasis is one of the most common human parasitic diseases whose socioeconomic impact is only surpassed by malaria [2, 3]. Schistosomiasis is caused by blood flukes of the genus *Schistosoma*, which have a complex life cycle comprising a vertebrate host and an invertebrate host. *Schistosoma mansoni* is one of the species that infects humans [4–6] and the most common etiological agent for human schistosomiasis, causing more than 83 million human infections in 54 countries [7].

Schistosomiasis treatment relies almost exclusively on the anthelmintic Praziquantel (PZQ). However this drug does not prevent reinfection and, with large-scale control programs promoting the extensive use of PZQ for more than 20 years in some African nations, concern regarding the selection of drug resistant parasites has been raised [8–10].

Resistance to PZQ is defined as the genetically transmitted loss of susceptibility in worm populations that were previously susceptible to PZQ. In this process, chemotherapy selectively removes susceptible individuals from the genetically heterogeneous populations leading to an increase of individuals carrying genotypic determinants conferring drug resistance that are passed to the offspring. After several generations, a large number of worms within the population survive following treatment [10].

*In vivo* artificial selection in mice has previously produced PZQ resistant lines of *S*. *mansoni* in only two generations after repeated exposure to sub-lethal doses of the drug [9], demonstrating that resistance is more than an hypothesis. Low cure rates in response to PZQ emerged 10–15 years ago after mass scale use in countries like Egypt and Senegal [11, 12]. Worms from the non-cured patients were repeatedly less susceptible to PZQ when tested in a mouse model [13]. Worm genetic determinants for resistance led to PZQ failure, although host factors, among other factors, were also considered to have contributed to PZQ failure such as heavy worm burdens and pre-patent infections [10, 13]. Difficulties in obtaining cure among travellers with schistosomiasis [13] further emphasized the need to maintain surveillance in order to avoid parasite spread to places where the intermediate host is present.

Chemotherapy failures in bacteria and cancer treatments have been associated to the activity of ATP-binding cassette (ABC) transport proteins [12, 14]. ABC-transport proteins are a large family of membrane proteins that have many multiple cellular functions including the transport of diverse compounds such as peptides, hormones, cholesterol and iron [14, 15]. Several members of this family also transport drugs, such as the P-glycoprotein (ABCB1, Pgp) and the multidrug resistance-associated proteins (MRPs and ABCCs), both reported to be involved in drug resistance by exporting drugs to the outside of parasites either by increased efflux activity or genetic over expression. PZQ is hypothesized to interact with Pgp-like or MRPs either as an efflux-substrate or as a competitor of transport mediated by the ABC-transport proteins of other efflux-substrates [16]. It has been demonstrated that the activity of efflux pumps (EP) of prokaryotes and eukaryotes can be inhibited by calcium channel blockers such as phenothiazines or Verapamil as they inhibit the transporter associated ATPases such as those that provide the energy for the activity of Pgp-like efflux pumps [16–19].

ABC transporter cDNAs that have been characterized in schistosomes includes *SmMDR2* [20], a *S*. *mansoni* orthologue of Pgp, and *SmMRP1* [21], a *S*. *mansoni* orthologue of MRP1. *SmMDR2* RNA is expressed at higher levels in female parasites than in males [20, 22], while males express higher *SmMRP1* RNA levels than females [21]. Notably, adults of *S*. *mansoni* up regulate the expression of both of these transporters in response to PZQ. Furthermore, higher basal levels of both *SmMDR2* and *SmMRP1* correlate with reduced PZQ susceptibility [21, 22], and PZQ inhibition activity, likely also a substrate of SmMDR2 [19]. Based on these findings, Kasinathan and colleagues have hypothesized that Schistosoma MDR transporters may be modulating the responsiveness of parasites to PZQ [18].

In this study, we have selected from a fully susceptible parasite strain (Belo Horizonte, Brazil line), by stepwise drug pressure, a *S*. *mansoni* variant strain that is stably resistant to PZQ. Our resistant parasite variant strain, obtained from infected mice, tolerates up to 1200 mg PZQ/kg of mouse body weight and is isogenic to its parental fully susceptible counterpart, except for the genetic determinants accounting for the PZQ-drug resistance phenotype. This resistant parasitic strain enabled us to further test the hypothesis that efflux pumps play an important role in the development of the PZQ drug resistance phenotype in *S*. *mansoni*. Therefore, the aim of this study was to evaluate the role of efflux pumps in *Schistosoma mansoni* PZQ resistance phenotype, by comparing the efflux pumps activity in the PZQ-susceptible and the PZQ-resistant parasite strains, upon exposure to a compound known to inhibit eukaryotic efflux pumps—Verapamil. A new methodology was also developed allowing the study, on a real time basis, of the transport of the universal efflux substrate ethidium bromide (EtBr) and to correlate the efflux inhibitory effects with the resistant variant, which over-expresses Pgp-like efflux-pumps demonstrating their important role on PQZ resistance in *S*. *mansoni*.

## Materials and Methods

### Reagents

The inhibitor Verapamil (Verap), Ethidium Bromide (EtBr) and Calcium Chloride (CaCl_2_) were purchased from Sigma-Aldrich (St. Louis, MO, USA). Praziquantel was purchased from Merck & Co. (Kenilworth, NJ, USA) and dissolved in Dimethyl Sulfoxide (DMSO) from Sigma-Aldrich, used for stock solutions, which were subsequently diluted to an appropriate concentration in culture media. All solutions were prepared in distilled, sterile water, on the day of the experiments.

### Animal Model

The PZQ resistant parasitic strain of *S*. *mansoni* was developed using *Mus musculus* CD1 line males, approximately eight weeks old. CD1 is considered the animal model of choice for *S*. *mansoni* infection, because it is a good host for this parasite mimicking the S. *mansoni* human infection [23].

The mice used, weighting around 20 g, were obtained from the animal breading facilities of Instituto de Higiene e Medicina Tropical/Universidade Nova de Lisboa (IHMT/UNL). They were kept in appropriate conditions of temperature (± 21°C), humidity (45–55%) and light (12-hour cycles of light/darkness). The infection occurred by exposing mice tails to about 100 cercariae of *S*. *mansoni* each, thus mice infection occurred by natural transdermal penetration of the cercariae.

### Parasite isolation

In this study we used a *S*. *mansoni* BH line (from Belo Horizonte, Brazil), susceptible to PZQ. This parasitic line is routinely kept in our group at IHMT/UNL, using *Biomphalaria glabrata* as intermediate host.

Our stable PZQ resistant parasite strain was obtained from the BH line submitted to various steps of PZQ continuous drug pressure, starting with 300 mg/kg and finishing with 1200 mg/kg of PZQ. Cioli and colleagues [13] demonstrated that in the murine model we can define a line of *S*. *mansoni* as resistant if it has a DL_50_ greater than 100 mg/kg, therefore our variant strain resists to 12 times this value.

**Fig 1:**
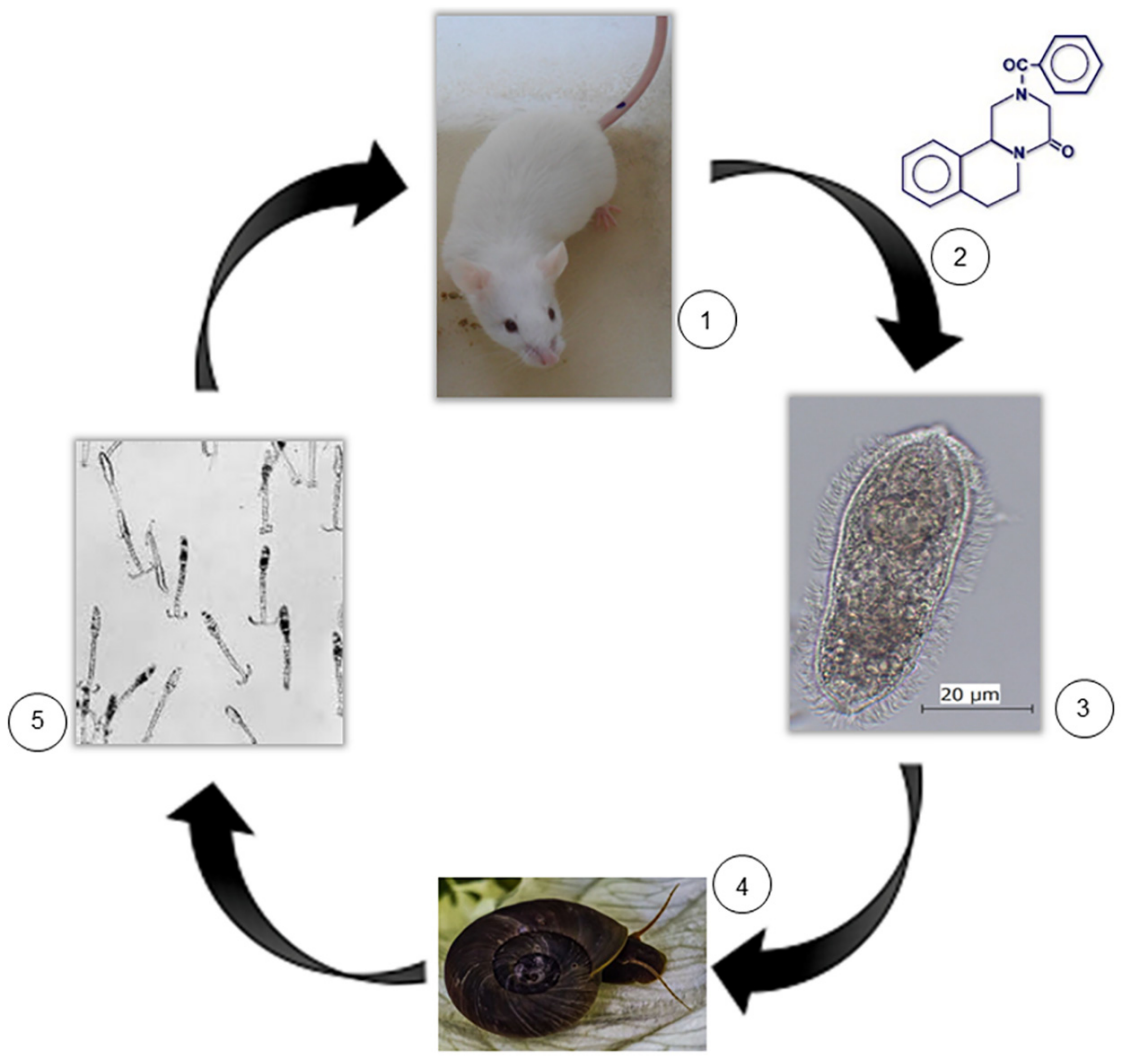
Selection of *S*. *mansoni* PZQ-resistant strain. This selection was carried out under continuous PZQ increased pressure using CD1 mice over several passages. 1—Transcutaneous infection of mice with ~100 cercariae; 2 –Oral administration of PZQ after infection confirmation by the presence of eggs in the faeces (± 60 dpi); 3 –Mice were euthanized to collect adult worms and miracidia (eggs in faeces) (± 75 dpi); 4—*B*. *glabrata* snails were infected with miracidia released from eggs; 5—Cercariae were released from snails (± 45 dpi).

Infected CD1 mice were checked approximately 60 days post parasite infection by Kato-Katz procedure; if eggs were found in faeces, mice were then treated orally with PZQ solution at appropriate dosage. If, on day 15, post PZQ treatment, viable eggs (verified by live miracidia inside the eggs and Kato-Katz procedure) continued to be eliminated, mice were euthanized and miracidia present in the liver were used to subsequently infect *B*. *glabrata* snails. Once *B*. *glabrata* snails start eliminating *S*. *mansoni* cercariae (30 to 60 days after snail infection), new CD1 mice were re-infected and the previous procedure was repeated, continuing the PZQ- resistant strain selection *in vivo* (Fig 1).

**Fig 2:**
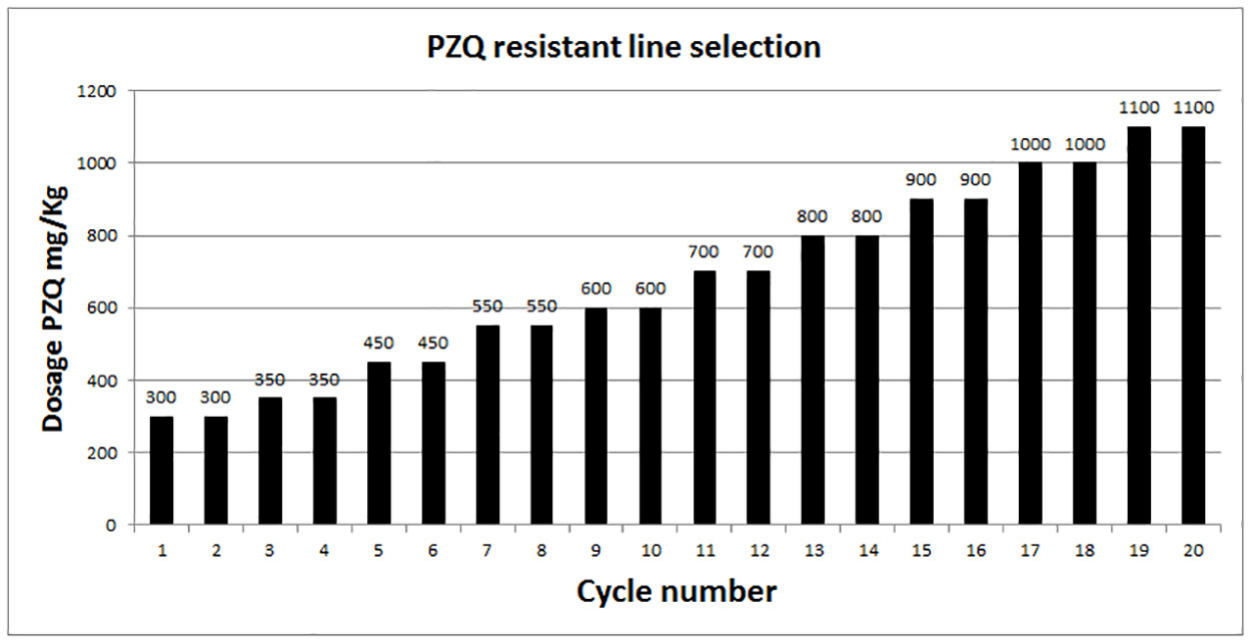
Schematic cartoon of PZQ dosages during the selection procedure for the *S*. *mansoni* PZQ-resistant strain. The parasite from BH susceptible strain was submitted to various steps of PZQ pressure, and the dosage was increased along the cycle number of passages.

PZQ dosage was increased every two passages as shown in Fig 2.

Adult worms (8–10 weeks post-infection) were collected by liver-perfusion, as described by Lewis and colleagues [24], and maintained in saline solution for the EtBr efflux assay or in RPMI medium (Sigma-Aldrich) for the *ex vivo* PZQ susceptibility assays.

### Ethidium bromide efflux assay

**Fig 3:**
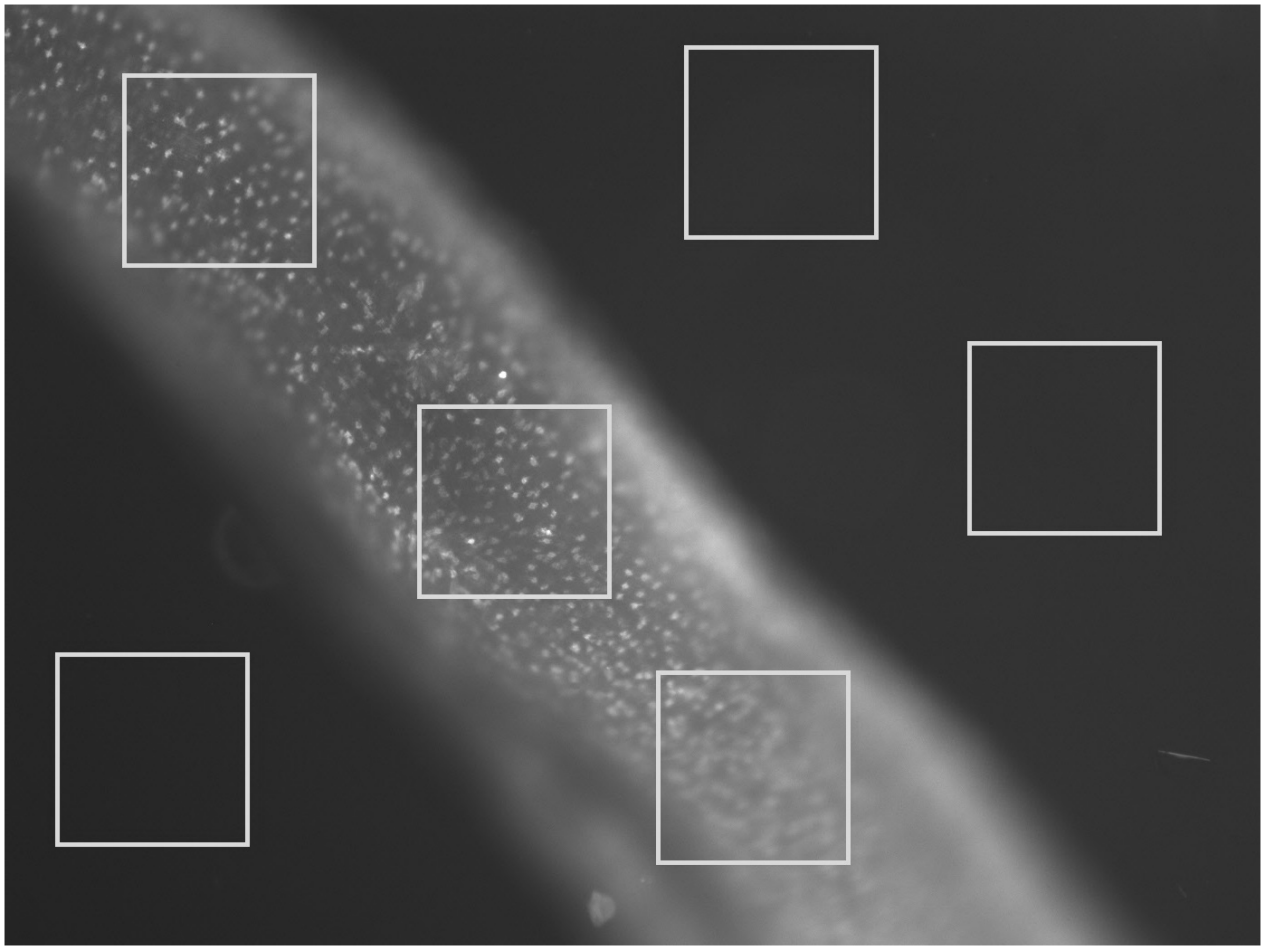
Schematic representation of the worm areas analysed by ImageJ. Fluorescence quantification was made in three defined regions, of the same size, corresponding to the worm central section (below the cecum ramification), of each worm and fluorescence intensity within each region was quantified using ImageJ software (imagej.nih.gov) and background intensity was subtracted.

Ethidium bromide efflux assay was performed with the objective of comparing the efflux pumps activity between males of both PZQ-susceptible and PZQ-resistant parasite strains as described by Viveiros and colleagues, adapted in this study for the assessment of parasite efflux activity [25]. Verapamil (2.2 μM and 4.4 μM), known as an inhibitor of ABCB1 (Pgp) efflux pump activity was used as EtBr efflux inhibitor at concentrations that did not compromise viability. EtBr concentration was previously optimized for each strain of adult worms in order to determine the lowest concentration which reflects the balance between EtBr accumulation by influx (passive diffusion) and extrusion by active efflux during the 35 minutes of the assay (EtBr influx-efflux steady-state whose accumulation (fluorescent signal) inside the worms is above the lowest signal detectable by the fluorescence microscope). This ensures that the observed increase of accumulation of EtBr during the 35 minutes of the assay is due to inhibition of efflux pumps that promotes increased accumulation of the fluorophore inside the worms [25]. To measure the time-curve of increased EtBr accumulation promoted by the inhibitor Verapamil, our EtBr control group were worms incubated with the same concentration of EtBr in the absence of Verapamil. All experiments were carried out in triplicate with three worms each (n = 9). For quantification of fluorescence, three areas, of each worm, of the same size, of the worm central section (below the cecum ramification), (as shown in Fig 3), were defined and fluorescence intensity was measured and quantified using ImageJ software (imagej.nih.gov) and background intensity was subtracted. Thus, each time-point of relative fluorescence in each assay corresponds to the mean of EtBr fluorescence (n = 9) that remains accumulated per unit of time that we compare to the EtBr control group (no inhibitor) [25].

After collecting parasites (as described before), they were separated by sex, and only males were used for this experiment since EtBr binds non-specifically to the blood present in the female’s intestine, thus defaulting the experiment. 24-well culture plates were prepared using RPMI–1640 culture medium, 200 mM L-glutamine, 10 mM HEPES, 24 mM de NaHCO_3_, 10000 UI of penicillin and 10 mg/mL of streptomycin were from Sigma-Aldrich, pH 7 and supplemented with 15% fetal bovine serum and three parasites were added on each well for each studied group. Parasites were incubated overnight at 37°C in a 5% CO_2_ atmosphere to recover from stress caused by liver perfusion. After this period, the worms were washed twice with saline solution to clean any traces of culture medium. The worms were then exposed to the inhibitor for one hour in the previous-mentioned concentrations, after which EtBr was added (0.6 μM) and parasites were observed under fluorescence microscopy (Zeiss, Axioskop HBO50) for a maximum of 35 min and pictures were taken every 2 min. After this period 1 mM of Calcium Chloride was added to reverse the inhibitory effect of Verapamil, pictures were taken every two minutes for 35 min, of all worms at the same exact position, magnification and fluorescence intensity for overall analysis of the assays. Three control groups were used: 1—Without Verapamil, 2—Without EtBr and 3—Without both Verapamil and EtBr (negative control). Fluorescence was quantified using the ImageJ software and compared between different groups.

### *Ex vivo* PZQ susceptibility assay

An *ex vivo* assay was devised to assess the susceptibility of adult worms of *S*. *mansoni* from both PZQ-susceptible and PZQ-resistant parasite strains, in the presence and absence of Verapamil, to ascertain the involvement of Pgp-like efflux pumps in the PZQ resistance phenotype.

Parasites were collected as previously described and separated by sex. 24-well culture plates were prepared as described in the previous section and various concentrations of PZQ and Verapamil were used in this susceptibility assay (Table 1), five worms were kept in each well and the same concentration of drug and inhibitor was used in two wells of the same plate. The experiment was done in triplicate, with at least 30 worms used for each concentration of drug and inhibitor. After adding Verapamil and/or PZQ, parasites were incubated for another 24 hours at 37°C in a 5% CO_2_ atmosphere after which the medium was switched for a drug free medium and kept for another 48 h. Parasites were observed every 12 h and the culture medium was changed after each observation. Parasites that did not present any movement after being observed at the microscope for a period of 2 min were considered dead. Lethal dosages were calculated using the software SPSS20^®^ for Windows using Probit regression model with a 95% confidence. The lethal dosages obtained were used for graphical construction design, using GraphPad Prism software.

**Table 1:** PZQ and Verapamil concentrations used for the *ex vivo* PZQ susceptibility assay.

Parasite strains	Parasite Sex	Verapamil (μM)	PZQ (μM)
Susceptible	Males	0.0	0–25.6
		0.2	0–25.6
		1.1	0–25.6
	Females	0.0	0–288.1
		4.4	0–288.1
Resistant	Males	0.0	0–128.0
		1.1	0–64.0
		2.2	0–48.0
		4.4	0–48.0
		8.8	0–32.0
	Females	0.0	0–2880.9
		8.8	0–2880.9

### RNA extraction and real-time qRT-PCR

Total RNA was extracted using Trizol (Invitrogen) from quick-frozen worms and then treated with DNase from Ambion according to the manufacturer’s instructions. Real-time reverse-transcriptase polymerase chain reaction (RT-PCR) was performed using the PerfectaSYBR Green SuperMix for iQ from Quanta Biosciences on an Opticon Real-Time PCR detection system from BioRad, according to the manufacturer’s recommendations. *Schistosoma mansoni 18S* (*Sm18s*) ribosomal RNA of each group was used as a reference gene in these experiments. Primers used for the amplification of *SmMDR2* gene were *SmMDR2* F (5’-TCTGACAATCGACCTGGTG–3’) and *SmMDR2* R (5’-CCAAGGAAGCAATGACTAAAAC–3’) and for *Sm18S* gene the primers were *Sm18S* F (5’-AGGAATTGACGGAAGGGCAC–3’) and *Sm18S* R 5’ACCACCCACCGAATCAAGAAAG–3’) [21]. For quantitative measurements, data was analysed using the 2^-ΔΔCt^ method [26] to determine the relative expression ratio between target gene (*SmMDR2*) and reference housekeeping gene (*Sm18S*), with appropriate calibrators and corrections for amplification efficiency. Three biological and technical replicates were used for the qRT-PCR experiments.

### Statistical Analysis

Data were expressed as mean ± standard deviation (SD), and tested for statistical significance using either ANOVA or unpaired *t*-tests. Probit regression model with a 95% confidence was used to calculate the lethal dosages, and the graphic construction was performed using GraphPad Prism 5.0 software.

### Ethics statement

This research project was reviewed and approved by the Ethics Committee and Animal Welfare (CEBEA), Faculty of Veterinary Medicine, UTL (Ref. 0421/2013). Animals were maintained and handled in accordance with National and European legislation (DL 276/2001 and DL 314/2003; 2010/63/EU adopted on 22 September 2010), with regard to the protection and animal welfare, and all procedures were performed according to National and European legislation.

## Results

### Ethidium bromide efflux assay

**Fig 4:**
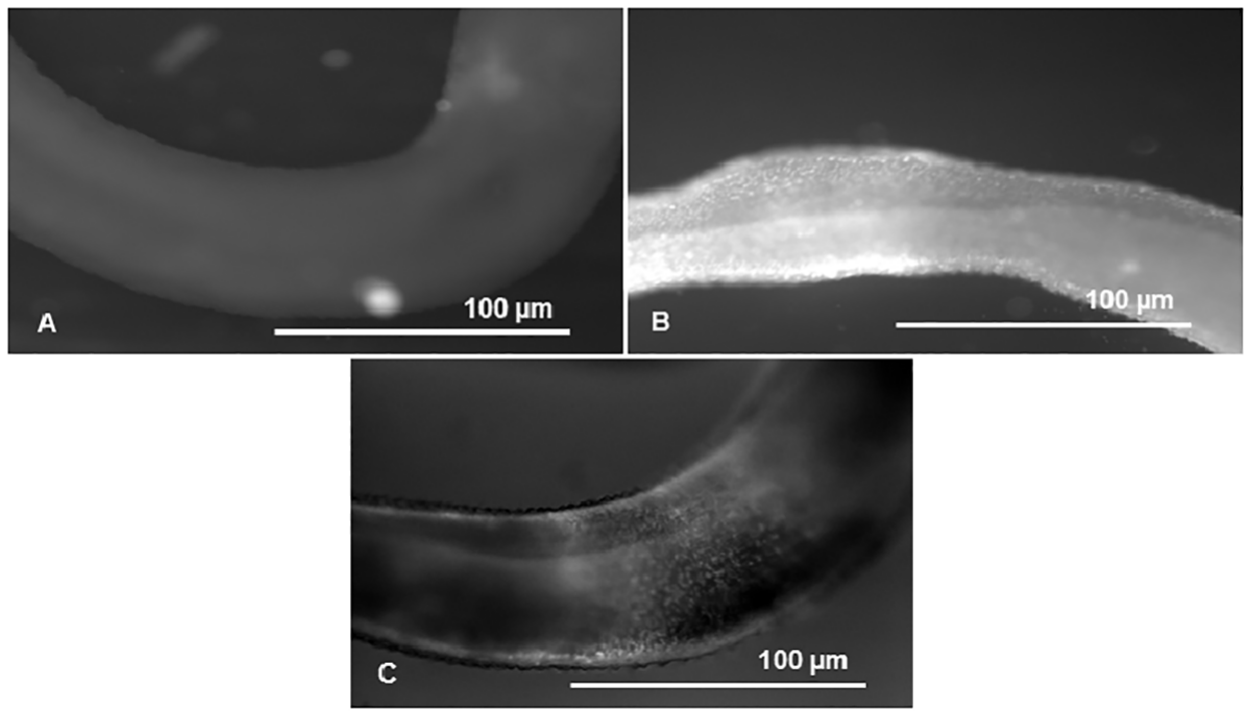
EtBr efflux assay in adult males of *S*. *mansoni* PZQ-susceptible strain. A) Control group—worms exposed to 0.6 μM of EtBr (20 min); B) worms exposed to 2.2 μM of Verapamil and 0.6 μM of EtBr (20 min); C) worms exposed to 2.2 μM of Verapamil, 0.6 μM of EtBr, and 1 mM de CaCl_2_ (35min).

Efflux pump activity was compared between PZQ-resistant and PZQ-susceptible adult males through fluorescence microscopy observation. EtBr is a common substrate to all efflux pumps, when outside the cells the signal is low, but when inside, the signal is amplified, and can be detected and quantified by time-course fluorescence spectroscopy [27]. Intracellular accumulation of EtBr after efflux inhibition by Verapamil was assessed by the increases in fluorescence intensity, using Image J software. As shown in Fig 4 in the susceptible variant strain after exposure to 2.2 μM of Verapamil, the efflux of EtBr was inhibited resulting in a clear increase of fluorescence, which decreased after the addition of Calcium Chloride. Verapamil is known to block the flow of calcium ions by binding to putative Ca^2+^ binding site [28–31], the addition of calcium revealed a reversing effect on the Verapamil inhibitory action on the efflux of EtBr, apparently restoring the function of the adult worms efflux pumps, thus reinforcing the hypothesis raised that the observed accumulation of EtBr in the adult males was due to the effect of this inhibitor on calcium-depended transporters, possibly by indirectly interfering with calcium dependent Pgp ATPases [28–31]. The control groups without EtBr showed viability and no intrinsic fluorescence was observed thus it is not represented. These findings are of importance considering that Pgp and MRPs are members of the “traffic ATPase” superfamily, which use the energy of ATP hydrolysis for maintaining their membrane transport function.

**Fig 5:**
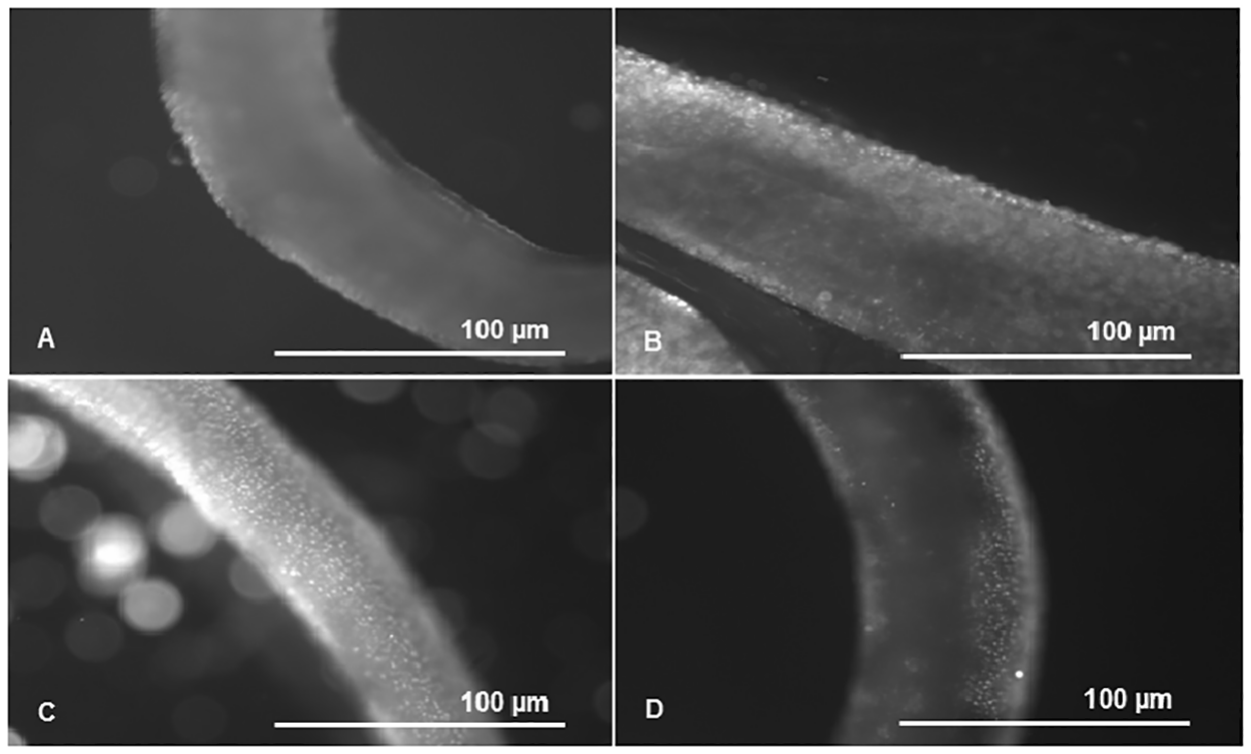
EtBr efflux assay in adult males of *S*. *mansoni* PZQ-resistant strain. A) Control group—worms exposed to 0.6 μM of EtBr (20 min); B) worms exposed to 2.2 μM of Verapamil and 0.6 μM of EtBr (20 min); C) worms exposed to 4.4 μM of Verapamil and 0.6 μM of EtBr (20 min); D) worms exposed to 4.4 μM of Verapamil, 0.6 μM of EtBr, and 1 mM de CaCl_2_ (35 min).

In the PZQ-resistant parasite strain, after exposure to 2.2 μM of Verapamil, there was an initial increase in fluorescence that later stabilized, showing EtBr accumulation levels lower than the PZQ-susceptible strain. A decrease in fluorescence was noticed after exposure to CaCl_2_. Only by exposing the PZQ-resistant strain to 4.4 μM of Verapamil, fluorescence levels reached levels similar to the susceptible parasites. (Fig 5).

**Fig 6:**
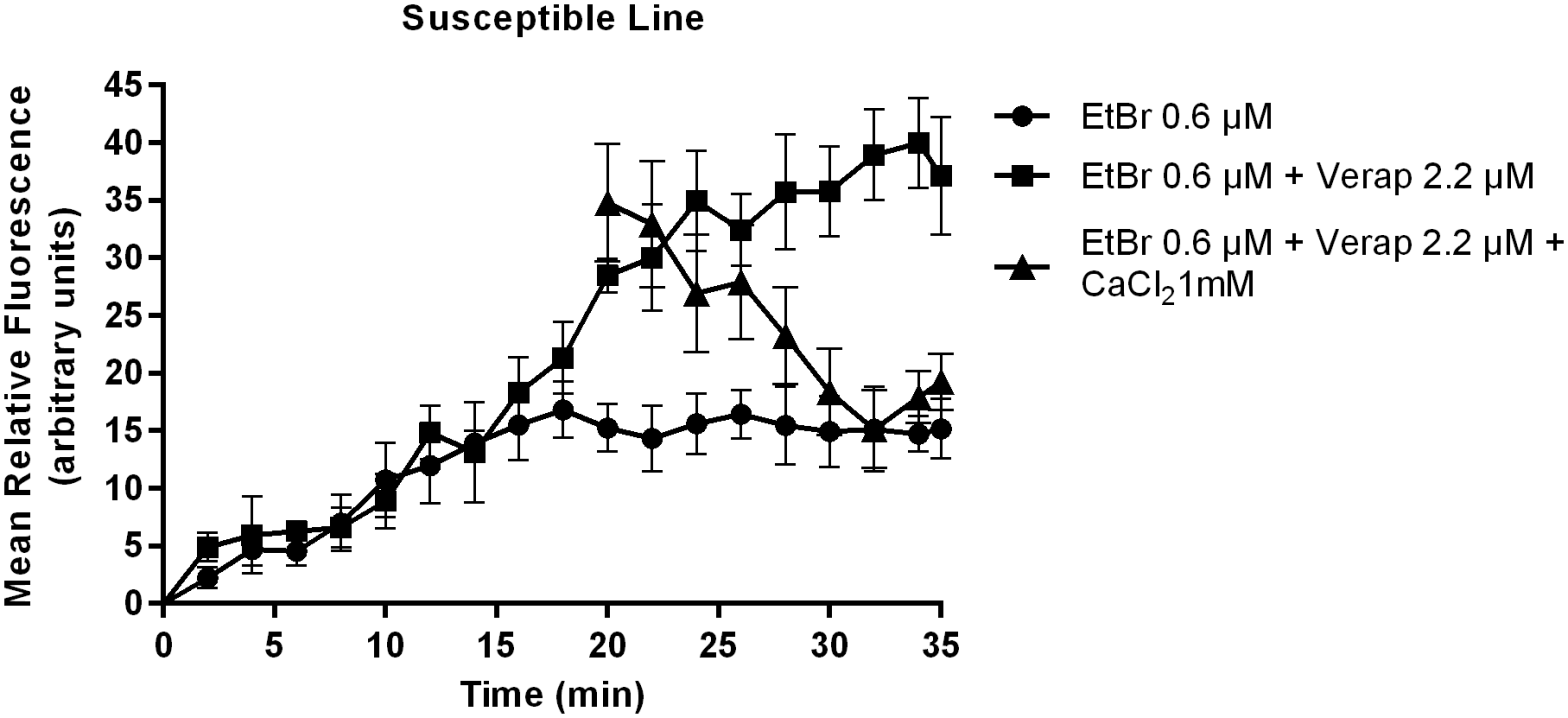
Variation in EtBr accumulation (Mean relative fluorescence). In the presence and absence of Verapamil and after the addition of CaCl_2_ in *S*. *mansoni* PZQ-susceptible adult males. Three worms were used for each group and the experiment was performed three times. Quantification measurements were made in three areas of the worm central section (below the cecum ramification) and background fluorescence was subtracted for each parasite at each time-point. The average measurement was calculated for each time-point. Data are expressed as mean fluorescence of the EtBr accumulated intracellularly over time.

**Fig 7:**
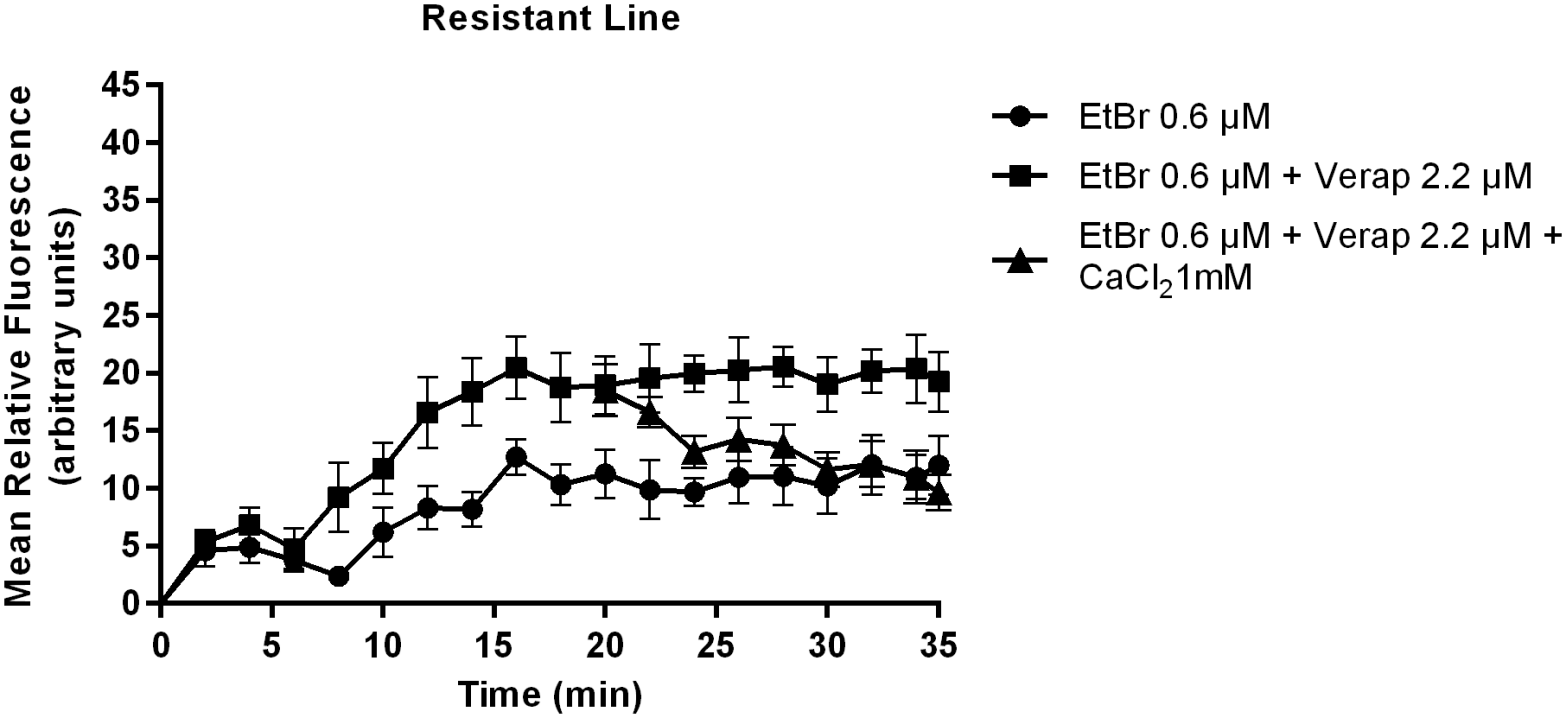
Variation in EtBr accumulation (Mean relative fluorescence). In the presence and absence of Verapamil and after CaCl_2_ addition in *S*. *mansoni* PZQ-resistant adult males. Three worms were used for each group and the experiment was performed three times. Quantification measurements were made in three areas of the worm central section (below the cecum ramification) and background fluorescence was subtracted for each parasite at each time-point. The average measurement was calculated for each time-point. Data are expressed as mean fluorescence of the EtBr accumulated intracellularly over time.

**Fig 8:**
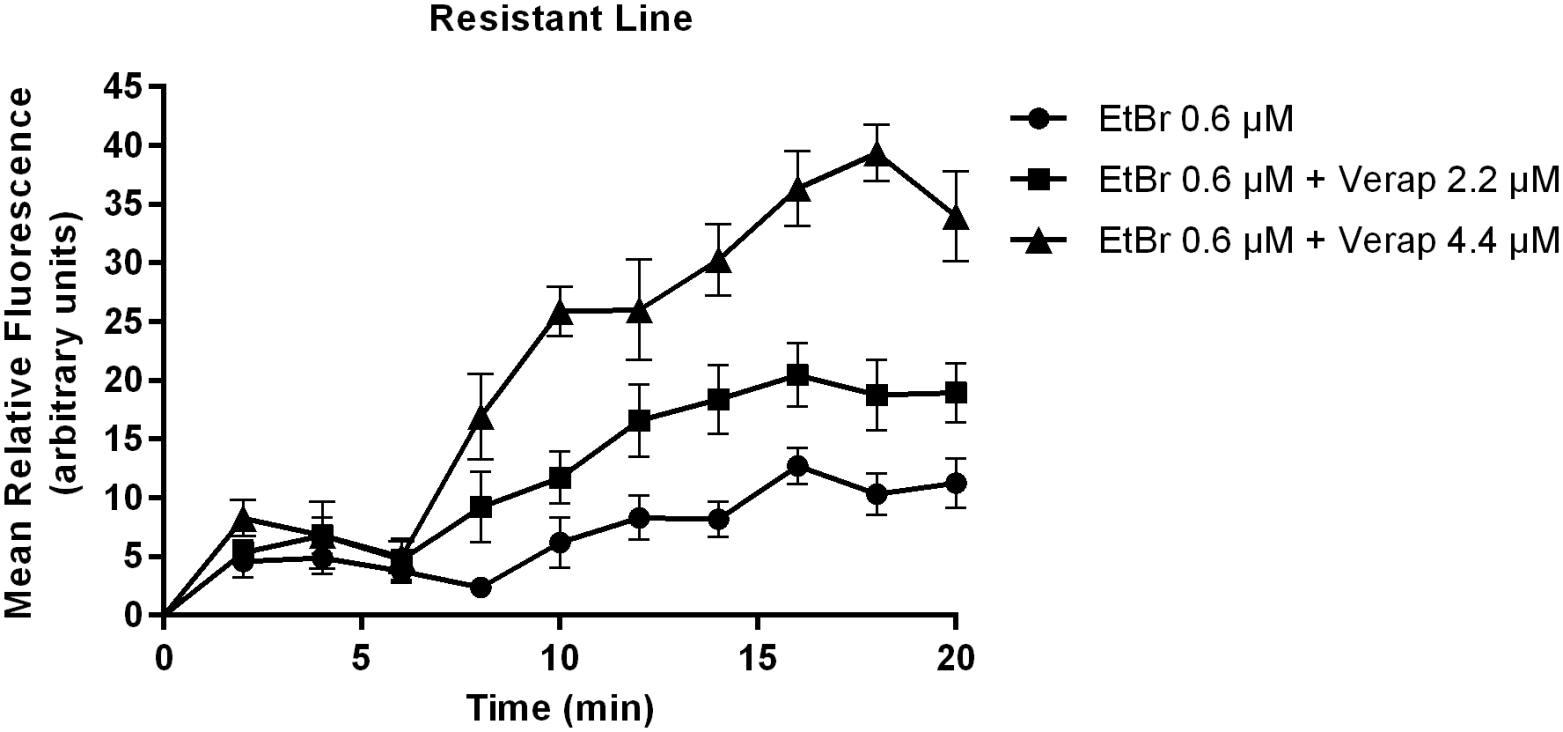
Variation in EtBr accumulation (Mean relative fluorescence). In the absence and presence of 2.2 μM and 4.4 μM of Verapamil in *S*. *mansoni* PZQ-resistant adult males. Three worms were used for each group and the experiment was performed three times. Quantification measurements were made in three areas of the worm central section (below the cecum ramification) and background fluorescence was subtracted for each parasite at each time-point. The average measurement was calculated for each time-point. Data are expressed as mean fluorescence of EtBr accumulated intracellularly over time.

As described in the Material and Methods section, throughout the efflux assays, fluorescence microscopy images were taken every 2 min for 35 min. Fluorescence was quantified in each picture in three areas of the worm central section (below the cecum ramification), (as shown in Fig 3), and background fluorescence was subtracted for each parasite (n = 9) at each time-points. The average was calculated and real-time efflux graphics were created to obtain an EtBr accumulation time course in presence and absence of Verapamil in both variant strains (Figs 6, 7 and 8). In the PZQ-susceptible parasite strain with the worms exposed to 2.2 μM of Verapamil, it was possible to observe a steady increase in the fluorescence over time, reaching approximately twice the mean relative fluorescence levels after 20 min, once compared to parasites not exposed to Verapamil. After the addition of 1 mM CaCl_2_ a sharp decrease in the fluorescence levels, reaching the same levels of those parasites not exposed to Verapamil (Fig 6).

In the PZQ-resistant strain, for the parasites exposed to 2.2 μM of Verapamil, there was an increase in fluorescence in the first 16 min, then maintaining a constant fluorescence over time at lower levels than the susceptible strain. No decrease in fluorescence was observed upon addition of 1 mM CaCl_2_ (Fig 7). Once exposed to 4.4 μM of Verapamil, the parasites showed a steady increase in the mean fluorescence over time (Fig 8). The PZQ-resistant parasite strain only showed fluorescence accumulation levels similar to the PZQ-susceptible strain when exposed to twice the concentration of Verapamil.

### *Ex vivo* PZQ susceptibility assay

#### PZQ-susceptible male worms

In the absence of Verapamil, adult males of the PZQ-susceptible strain achieved a 50% lethal dose (LD_50_) when exposed to 17.8 μM of PZQ; a lethal dose of 90% (LD_90_) when exposed to 24.2 μM of PZQ and a lethal dose of 99% (LD_99_) when exposed to 31.0 μM of PZQ.

In the presence of Verapamil, it was possible to observe a reduction in the amount of PZQ required to achieve the lethal dosages mentioned above. In the presence of 0.2 μM and 1.1 μM of Verapamil the LD_50_ was 15.1 μM and 12.5 μM of PZQ, respectively. LD_90_ was 20.6 μM and 16.9 μM of PZQ, and LD_99_ was 26.4 μM and 21.7 μM of PZQ, respectively (Table 2).

**Table 2:** Lethal doses of PZQ (LB—Lower Bound; UB—Upper Bound) calculated using Probit regression model with a 95% confidence, for *S*. *mansoni* PZQ-susceptible males in the presence of different concentrations of Verapamil.

Verapamil Concentration (μM)	Mortality (%)						
	1	10	30	50	70	90	99
	PZQ Concentration (μM)						
**0**	10.2 (LB– 8.37; UB– 11.21)	13.1 (LB– 10.53; UB– 15.00)	15.7 (LB– 13.41; UB– 17.70)	17.8 (LB– 15.62; UB– 20.15)	20.2 (LB– 17.89; UB– 23.34)	24.2 (LB– 21.21; UB– 29.58)	31.0 (LB– 26.14; UB– 40.11)
**0.2**	8.7 (LB– 6.11; UB– 10.53)	11.1 (LB– 8.73; UB– 12.94)	13.4 (LB– 11.16; UB– 15.21)	15.1 (LB– 13.06; UB– 17.24)	17.2 (LB– 15.05; UB– 19.83)	20.6 (LB– 17.99; UB– 24.83)	26.4 (LB– 22.32; UB– 35.26)
**1.1**	7.2 (LB– 5.06; UB– 8.66)	9.2 (LB– 7.22; UB– 10.64)	11.0 (LB– 9.52; UB– 12.52)	12.5 (LB– 10.78; UB– 14.21)	14.1 (LB– 12.41; UB– 16.37)	16.9 (LB– 14.82; UB– 20.60)	21.7 (LB– 18.26; UB– 29.37)

**Fig 9:**
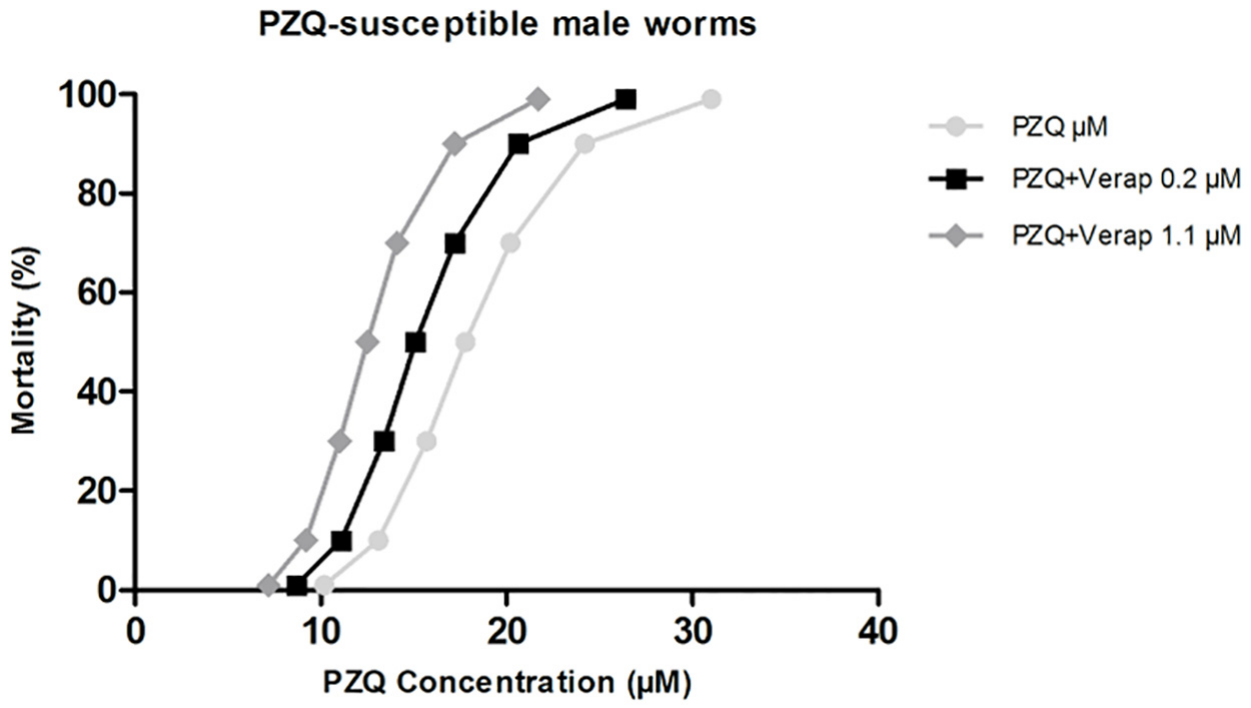
Mortality trends of *S*. *mansoni* adult males PZQ-susceptible exposed to PZQ in the presence of Verapamil. The mortality levels to increase concentrations of Verapamil (0.2 and 1.1 μM) are represented by survival curves. Additionally, the survival curve of parasites unexposed to Verapamil is also represented. The Probit regression model was used with a 95% of confidence.

The lethal dose values calculated using PZQ-susceptible parasite strain adult males were applied for the construction of mortality trend curves to get a better view of Verapamil effect on their susceptibility to PZQ (Fig 9). A decrease in the PZQ concentration required to achieve the same level of mortality was evident once compared to parasites not exposed to the inhibitor.

#### PZQ-resistant male worms

In the absence of Verapamil, male worms of the PZQ-resistant strain achieved the LD_50_ when exposed to 65.2 μM of PZQ, LD_90_ when exposed to 98.1 μM and the LD_99_ when exposed to 137.0 μM of PZQ.

When exposed to a non-toxic concentration of Verapamil, it was possible to observe a reduction in the amount of PZQ required to achieve the lethal dosages mentioned above. In the presence of four different concentrations of Verapamil (1.1 μM, 2.2 μM, 4.4 μM, and 8.8 μM), the PZQ lethal dose decreased significantly: LD_50_ concentrations of PZQ was 33.9 μM, 19.7 μM, 5.1 μM and 3.6 μM, LD_90_ was 52.4 μM, 37.5 μM, 19.8 μM and 12.8 μM and the LD_99_ was 74.7 μM, 63.2 μM, 59.8 μM and 35.9 μM, for each of the four concentrations of inhibitor used (Table 3).

**Table 3:** Lethal doses of PZQ (LB—Lower Bound; UB—Upper Bound) calculated using Probit regression model with a 95% confidence, for *S*. *mansoni* PZQ-resistant parasite strain males in the presence of various concentrations of Verapamil.

Verapamil Concentration (μM)	Mortality (%)						
	1	10	30	50	70	90	99
	PZQ Concentration (μM)						
**0**	30.98 (LB– 22.30; UB– 37.51)	43.27 (LB– 35.15; UB– 49.15)	55.11 (LB– 48.34; UB– 60.44)	65.16 (LB– 59.27; UB– 70.95)	77.05 (LB– 70.76; UB– 85.51)	98.14 (LB– 87.99; UB– 116.32)	137.03 (LB– 115.75; UB– 182.62)
**1.1**	15.39 (LB– 9.91; UB– 19.23)	21.95 (LB– 15.62; UB– 28.42)	28.38 (LB– 25.42; UB– 31.37)	33.92 (LB– 27.81; UB– 37.76)	40.53 (LB– 38.20; UB– 52.21)	52.41 (LB– 45.26; UB– 58.07)	74.74 (LB– 64.41; UB– 84.63)
**2.2**	6.16 (LB –1.46; UB– 9.83)	10.38 (LB– 4.34; UB– 14.20)	15.17 (LB– 9.21; UB– 19.18)	19.72 (LB– 14.55; UB– 25.21)	25.64 (LB– 20.38; UB– 37.35)	37.46 (LB– 28.43; UB– 76.87)	63.20 (LB– 41.46; UB– 88.66)
**4.4**	0.44 (LB– 0.08; UB– 1.02)	1.33 (LB– 0.42; UB– 2.35)	2.95 (LB– 1.42; UB– 4.37)	5.13 (LB– 3.18; UB– 6.93)	8.92 (LB– 6.56; UB– 11.95)	19.85 (LB– 14.44; UB– 34.00)	59.81 (LB– 34.70; UB– 67.70)
**8.8**	0.36 (LB– 0.02; UB– 0.94)	1.01 (LB– 0.38; UB– 1.86)	2.15 (LB– 1.25; UB– 3.00)	3.60 (LB– 2.98; UB– 4.98)	6.05 (LB– 5.35; UB– 9.02)	12.79 (LB– 10.18; UB– 17.60)	35.94 (LB– 22.72; UB– 40.65)

**Fig 10:**
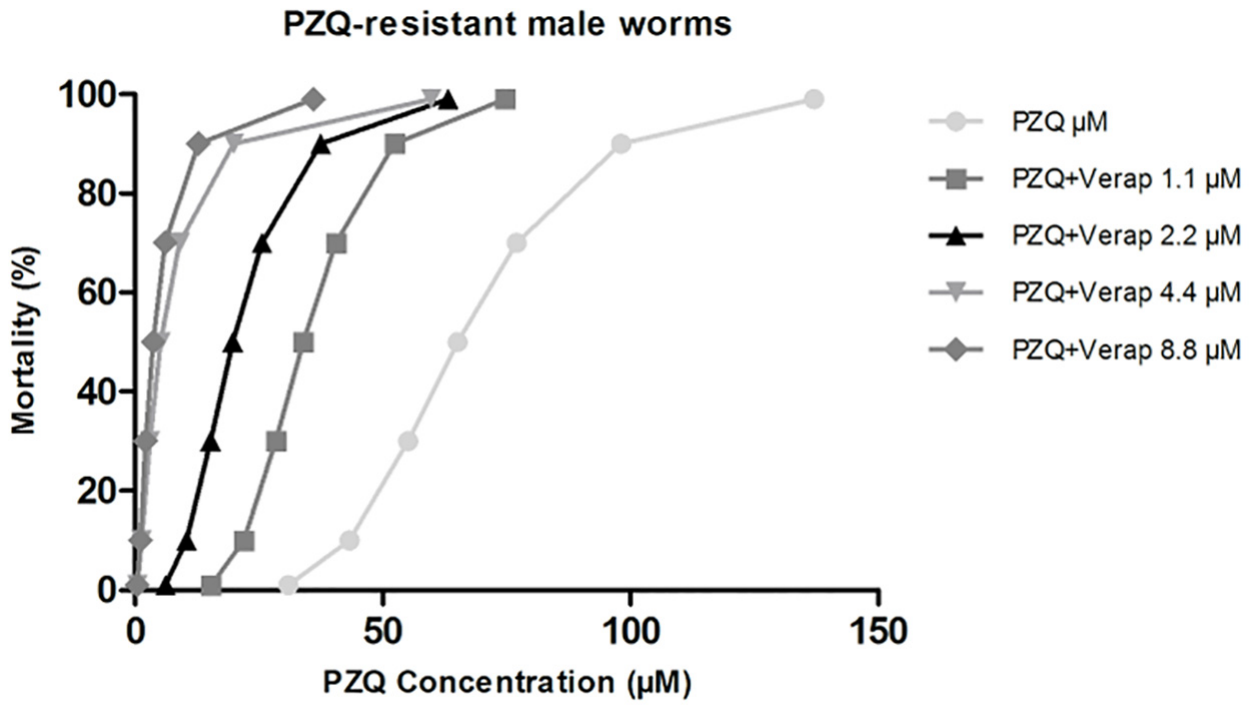
Mortality trends of *S*. *mansoni* adult males PZQ-resistant exposed to PZQ in the presence of Verapamil. The mortality levels to increase concentrations of Verapamil (1.1–8.8 μM) are represented by survival curves. Additionally, the survival curve of parasites unexposed to Verapamil is also represented. The Probit regression model was used with a 95% of confidence.

The lethal PZQ dose values for PZQ-resistant males when exposed to different concentrations of Verapamil were plotted in a mortality dose dependent curve (Fig 10) showing the effect of Verap on the susceptibility to PZQ in this variant strain. Once again, in the presence of the efflux inhibitor (Verapamil), a decrease in the PZQ concentration required to achieve the same level of mortality was observed, compared to parasites not exposed to this inhibitor. In the presence of 1.1 μM of Verapamil, the lowest concentration tested in this strain, the PZQ lethal concentrations were twice as low compared to the ones obtained for the group not exposed to the inhibitor. Overall, it was demonstrated that the drug-resistant strain reduces or reverts its resistance to PZQ in the presence of Verapamil obtaining LD values close to or even lower than those obtained for the susceptible variant strain.

#### PZQ-susceptible female worms

**Fig 11:**
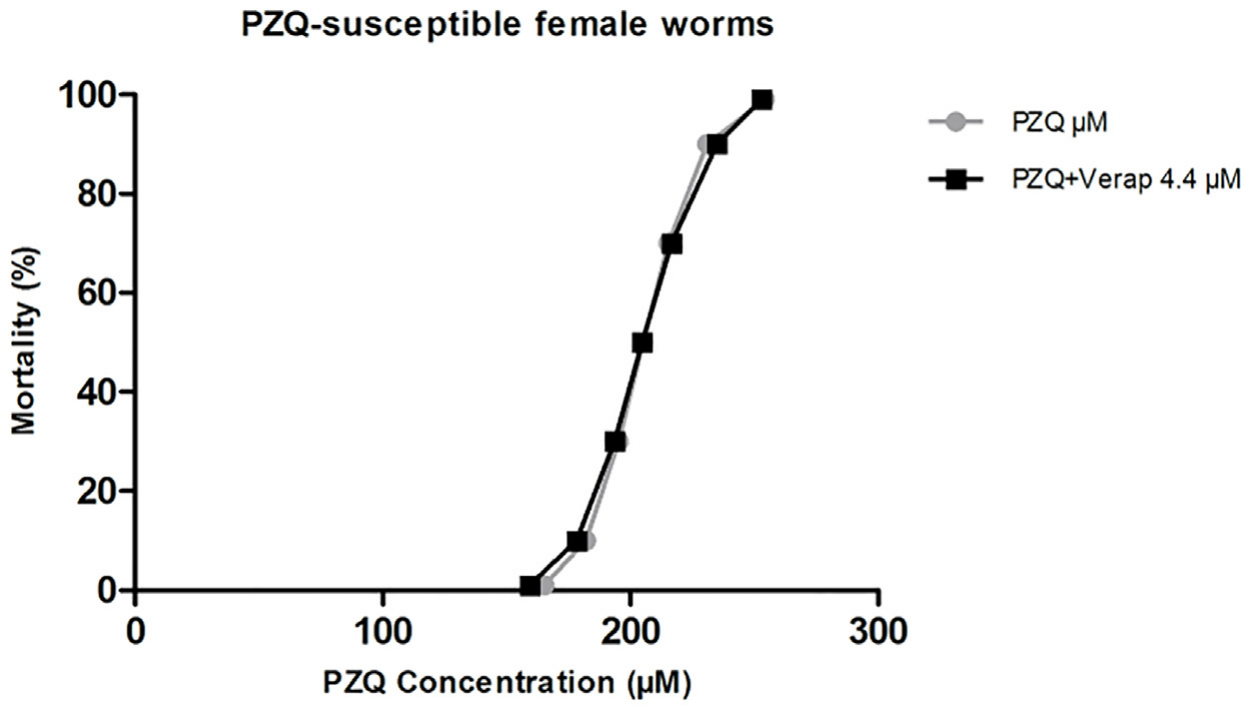
Mortality trends *S*. *mansoni* adult females PZQ-susceptible exposed to PZQ in the presence of Verapamil. The mortality levels to 4.4 μM Verap is represented by a survival curve. Additionally, the survival curve of parasites unexposed to Verapamil is also represented. The Probit regression model was used with a 95% of confidence.

In the absence of Verapamil, susceptible strain females presented a LD_50_ of 205.02 μM, a LD_90_ of 230.84 μM, and a LD_99_ of 254.29 μM of PZQ (Table 4). When exposed to the highest concentration of Verapamil used in this study (4.4 μM), no differences in PZQ susceptibility were noticed (Fig 11). Our results put in evidence that PZQ-susceptible female worms are more resistant to PZQ than males from the resistant strain.

**Table 4:** Lethal doses of PZQ (LB—Lower Bound; UB—Upper Bound) calculated using Probit regression model with a 95% confidence, for *S*. *mansoni* PZQ-susceptible parasite strain females in the presence of different concentrations of Verapamil.

Verapamil Concentration (μM)	Mortality (%)						
	1	10	30	50	70	90	99
	PZQ Concentration (μM)						
**0**	165.29 (LB– 156.91; UB– 171.61)	182.08 (LB– 176.07; UB– 186.74)	195.30 (LB– 190.94; UB– 199.00)	205.02 (LB– 201.34; UB– 208.62)	215.21 (LB– 211.43; UB– 219.61)	230.84 (LB– 225.60; UB– 237.86)	254.29 (LB– 245.62; UB– 266.75)
**4.4**	159.22 (LB– 150.43; UB– 168.33)	178.33 (LB– 166.08; UB– 181.26)	193.52 (LB– 189.62; UB– 195.99)	204.76 (LB– 200.25; UB– 208.55)	216.62 (LB– 209.86; UB– 219.01)	234.90 (LB– 230.22; UB– 238.73)	252.98 (LB– 244.44; UB– 263.93)

Although the concentration of PZQ associated with female worm data seems high, PZQ used in vitro is not metabolized, and PZQ metabolites have shown a higher anti-schistosomal activity than pure unmetabolized PZQ (used *in vitro*) [32].

It should also be noticed that *Schistosoma* females have a much higher tolerance to PZQ than males as shown by Pica-Mattoccia and Cioli [33] and by Liang and colleagues [34]. The results we obtained for LD50 in females stays somewhere in between the ones obtained by these authors.

#### PZQ-resistant female worms

PZQ-resistant female worms were exposed to PZQ concentrations up to 2880.92 μM, in the presence and absence of Verapamil, and it was not possible to determine any lethal dosages. However, long-term effects of PZQ and the effects of the host immune system were not taken into account.

### Real-time PCR

**Fig 12:**
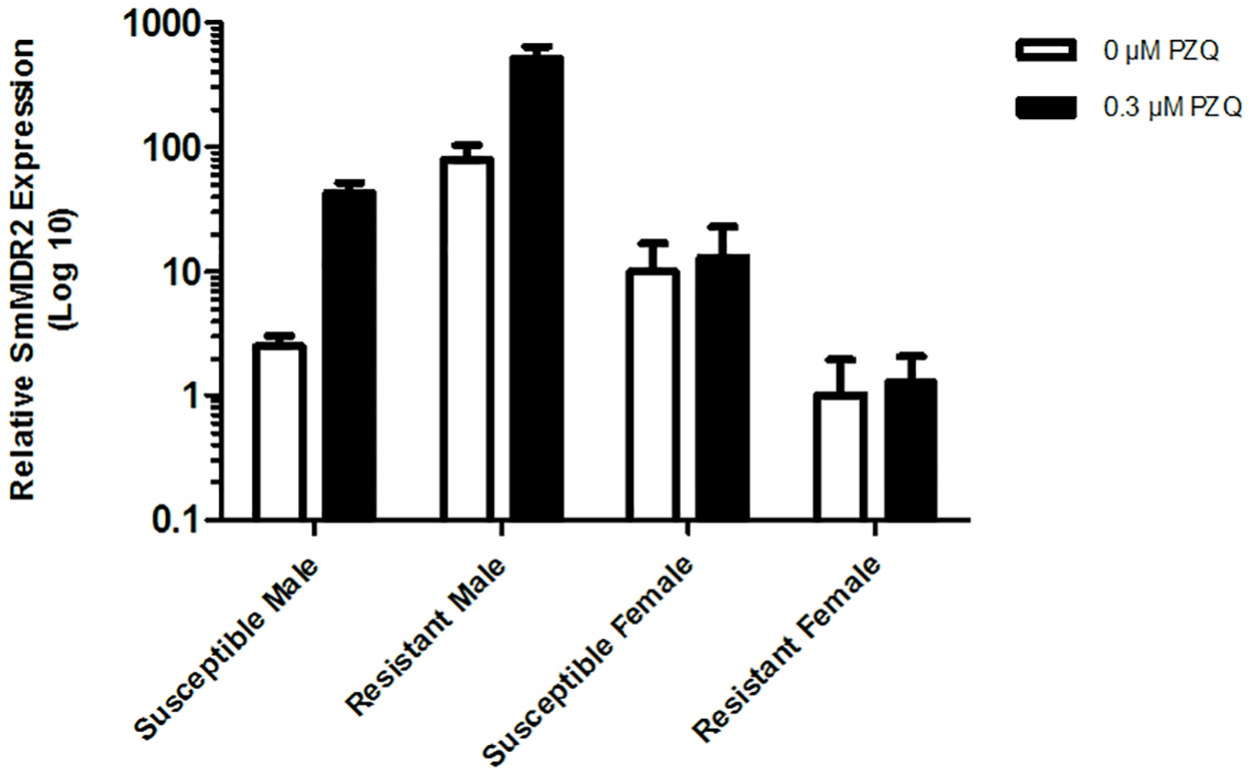
Relative expression level of *SmMDR2* RNA. In males and females of PZQ-susceptible and PZQ-resistant parasite strains in the presence and absence of PZQ. White bars—RNA from adult worms without exposure to PZQ, and black bars—RNA from adult worms after exposure to PZQ. The n-fold changes were determined by quantitative RT-PCR using *S*. *mansoni 18S* (*Sm18s*) RNA of each group was used as a reference gene. Differences of the relative level of *SmMDR2* between the groups was done using ANOVA, *p* < 0.05.

The relative expression levels of *SmMDR2* gene were assessed using a quantitative RT-PCR method. Adult worms of each PZQ-strain were separated by sex and compared. Parasites were also compared in the presence and absence of 0.3 μM of PZQ for 3 hours. As shown in Fig 12, when comparing PZQ-susceptible males and females, before exposure to PZQ, females showed a relative increase in the expression level of *SmMDR2* of approximately 4 times when compared to males (*p* < 0.05). When exposed to PZQ the expression level of *SmMDR2* in susceptible males increased 17 times when compared to the expression level of the same gene in the absence of PZQ in females (*p* < 0.05). As expected PZQ-resistant males showed, in the absence of PZQ, an increase in the expression level of *SmMDR2* of approximately 32 times when compared to PZQ-susceptible males (*p* < 0.05). Furthermore, after exposure to PZQ, *SmMDR2* expression level of PZQ-resistant males was approximately 6 times higher than in the absence of PZQ (*p* < 0.05). Finally, PZQ-resistant females showed no significant change in *SmMDR2* expression after exposure to PZQ (*p* > 0.05), and the expression was approximately 10 times lower than the PZQ-susceptible females (*p* < 0.05).

In the EtBr efflux assay, when observing both PZQ-susceptible and PZQ-resistant adult males, fluorescence levels in the absence of Verapamil, did not vary significantly, however when observing the expression of *SmMDR2* RNA through RT-PCR, there was a significant difference in *SmMDR2* expression. However we believe this could be explained by a higher sensitivity of RT-PCR.

## Discussion

We have selected, for the first time, to our knowledge, a stable PZQ resistant parasite strain that resists to 1200 mg/kg of PZQ. This strain was obtained by PZQ continuous drug pressure, and this PZQ resistant parasite strain was used to analyse the involvement of efflux pumps in the observed induced PZQ-drug resistance phenotype. Efflux pump activity of *S*. *mansoni* adult male worms, was observed and monitored by fluorescence microscopy using for the first time an adaptation of the semi-automated fluorometric methodology described by Viveiros and colleagues [25]. EtBr was used as a universal fluorescent substrate in the presence and absence of an efflux inhibitor—Verapamil—thus the emission of the accumulated fluorescence was monitored throughout sequential photographs, taken every 2 min, during a maximum period of 35 min. This assay was only possible to perform with adult males, because EtBr binds non-specifically to the blood present in the female’s intestine, turning impossible to distinguish differences of efflux-pump activity in the female worms [35].

In PZQ-susceptible adult males, the exposure to 2.2 μM of Verapamil led to a substantial increase in accumulated fluorescence suggesting that Verapamil is able to inhibit EtBr efflux in *S*. *mansoni* males of the susceptible strain. Reversal of this effect was possible after the addition of a non-toxic concentration of CaCl_2_, suggesting that CaCl_2_ has an important role in the mechanism responsible for reversing the efflux inhibitory effect of Verapamil in *Schistosoma* spp. This involvement of calcium ions has been previously described as anecdotic observations in the literature [28–31] with a possible relation between calcium homeostasis and Pgp mediated MDR reported by Sulová and co-authors [30]. At present, there is no scientifically accepted mechanism by which calcium reverses the effects of verapamil on Pgp activity but our novel observation raises questions that will be explored in future works, namely the connection between calcium homeostasis, Pgp activity and energy/ATP synthesis used for active transport of substrates [29, 30] In PZQ-resistant adult males EtBr accumulation was up to 2 times lower than the PZQ-sensitive males, when exposed to 2.2 μM of Verapamil. Only when PZQ-resistant adult males were exposed to 4.4 μM of Verapamil the intracellular accumulation of EtBr was similar to the susceptible variant strain. This suggests that males of the resistant strain have a higher number of transporters responsible for the EtBr efflux, which was further demonstrated by the quantitative RT-PCR results on the *SmMDR2* RNA expression level. Other authors have also shown an increased expression level of the *SmMDR2* RNA in PZQ resistant clinical isolates of *S*. *mansoni* [19, 21, 22, 36, 37]. Here in this paper we were able to demonstrate that there is an increase of Pgp-like efflux pumps activity in male worms from resistant strains which is in agreement with our results obtained by RT-PCR for parasites exposed for 3 hours with sub lethal concentrations of PZQ and correlation to its reduced susceptibility to PZQ. Messerli and colleagues [22] observed an increase of *SmMDR2* mRNA in females after being exposed to PZQ for 24–48h, which were not coincident with expression of SMDR2 protein. Despite the absence of the assays of *SmMDR2* expression at protein level our ex-vivo PZQ susceptibility assays suggests that Pgp-like proteins do not play a relevant role on PZQ-susceptibility in female worms.

The greatest advantage of our experimental model over other PZQ resistant parasites described in the literature is the fact that they are isogenic allowing comparing the influence of efflux pumps in PZQ resistant phenotype within the same genetic background. Therefore, it was possible to observe that the *S*. *mansoni* adult males variant resistant to PZQ presented an increased efflux pump activity suggesting that Pgp-like efflux pumps play an important role in PZQ-drug resistance in *S*. *mansoni*. In the EtBr efflux assay, when observing both PZQ-susceptible and PZQ-resistant adult males, fluorescence levels in the absence of Verapamil, resistant strain males showed lower levels of fluorescence. This could be explained by a higher number of EtBr efflux pumps in the resistant strain, which, is further reinforced when observing the expression of *SmMDR2* RNA, through RT-PCR, where a significant difference in *SmMDR2* expression can be observed. To further put in evidence that over-expression of efflux pumps is involved in PZQ acquired drug-resistance, an *ex vivo* assay, using both *S*. *mansoni* strains, was performed to assess the degree of susceptibility of the adult parasites to PZQ, in the presence and absence of Verapamil. When adult males of susceptible strain were exposed to Verapamil the PZQ concentration required to reach lethal dosages was lower than those observed in the absence of the inhibitor. Other authors have already reported that blocking the activity of the Pgp and MRPs transporters by Verapamil increases the pharmacological susceptibility of helminths such as *Caenorhabditis elegans*, *Haemonchus contortus*, and *Cooperia oncophora* to various anthelminthic drugs [38, 39]. For male worms of PZQ-resistant strain, in the presence of this efflux inhibitor, a lower PZQ concentration was required to achieve the same level of mortality compared to the same parasites not exposed to the inhibitor. In the presence of the lowest concentration of Verapamil tested in the resistant strain, PZQ lethal concentrations were twice as low as the ones obtained for the group not exposed to the inhibitor.

Overall, it was possible to observe that PZQ susceptibility of the PZQ-resistant strain, in the presence of Verapamil, has LD values close to or even lower than those obtained for the PZQ-susceptible strain. Ardelli and Prichard also showed that a *C*. *elegans* Ivermectin-resistant strain in the presence of Verapamil, presented an increased susceptibility to Ivermectin, suggesting an involvement of Pgp-like efflux pumps on this Ivermectin drug resistance phenotype [38]. Our results also suggest that, just as in the resistant strain of *C*. *elegans*, the adult males of our resistant strain have Pgp pumps involved in the drug resistance phenotype as demonstrated by the *SmMDR2* expression level analysis.

It is reported in literature collateral sensitivity (CS) of drug-resistant cancer cells to Verapamil [40–42], a phenomenon that might have happened in our *ex vivo* PZQ susceptibility assay in PZQ-resistant worms by a mechanism possibly linked to the expression of *SmMDR2*. This weakness, observed by PZQ LD50 obtained from resistant worms in the presence of increasing concentrations of Verapamil, can circumvent potential problems that might be associated with adjuvant therapy using EPIs during standard therapy with PZQ, where the main objective is to treat patients by killing all the worms (susceptible and resistant worms) without causing side effects. Also, CS opens a new approach for the identification of new re-sensitizing compounds in the management of PZQ resistance and to elucidate the mechanisms involved.

In contrast, female adult worms did not present any difference in the observed lethal dosages of PZQ, in the presence or in absence of Verapamil, which gives an indication that the activity of Pgp-like efflux pumps is not involved in PZQ susceptibility of adult female worms. Furthermore, the values obtained for lethal dosage suggests a higher tolerance of female worms to PZQ. This higher tolerance has already been described in other reports [21, 33, 36] in which adult female worms tolerate considerably higher concentrations of PZQ than adult males both *ex vivo* and *in vivo* [9, 11, 12, 43, 44, 45].

Previous studies regarding drug resistance, have already presented evidence of an increased tolerance to PZQ in male worms [19, 21, 22, 36, 37, 46]. It should be noted that when using *in vivo* and *ex vivo* assays the interaction between the effects caused by the drug and those caused by the host immune system on the parasite are not taken in consideration [33, 47, 48]. In conclusion, our work describes for the first time, the application of a successful methodology previously applied in bacteria and cancer cells, using the universal efflux pump substrate EtBr, for the evaluation of drug transporter systems on *S*. *mansoni* adult worms as a multicellular cell model using an *ex vivo* assay. The methodology used have demonstrated the involvement of adult male schistosomes Pgp-like transporters *SmMDR2* in PZQ drug resistance phenotype, evidenced by the fact that lower doses of Verapamil successfully reverted PZQ drug resistance when using sub lethal concentrations of PZQ. World Health Organization warns about the possible emergence of *Schistosoma* spp. populations that are resistant to PZQ thus recommending continued vigilance [49]. Therefore, studies on genetic resistance mechanisms against PZQ are of extreme importance to understand the potential mechanism(s) of resistance/increase tolerance to PZQ, contributing to the development of new drugs and the delineation of new strategies for schistosomiasis control.
